# Novel cerebrospinal fluid biomarkers correlating with shunt responsiveness in patients with idiopathic normal pressure hydrocephalus

**DOI:** 10.1186/s12987-023-00440-5

**Published:** 2023-06-05

**Authors:** Sophia Weiner, Antti Junkkari, Mathias Sauer, Antti Luikku, Tuomas Rauramaa, Tarja Kokkola, Sanna-Kaisa Herukka, Kaj Blennow, Henrik Zetterberg, Ville Leinonen, Johan Gobom

**Affiliations:** 1 Department of Psychiatry and Neurochemistry, Institute of Neuroscience and Physiology, The Sahlgrenska Academy at the University of Gothenburg, Mölndal, Sweden; 2 Department of Neurosurgery, NeuroCenter, Kuopio University Hospital and Neurosurgery, Institute of Clinical Medicine, University of Eastern Finland, Kuopio, Finland; 3 Clinical Neurochemistry Laboratory, Sahlgrenska University Hospital, Mölndal, Sweden; 4 Department of Pathology, Kuopio University Hospital and University of Eastern Finland, Kuopio, Finland; 5 Department of Neurology, Kuopio University Hospital and University of Eastern Finland, Kuopio, Finland; 6 Department of Neurodegenerative Disease, UCL Institute of Neurology, Queen Square, London, UK; 7 UK Dementia Research Institute, London, UK; 8 Hong Kong Center for Neurodegenerative Diseases, Hong Kong, China; 9 Wisconsin Alzheimer’s Disease Research Center, University of Wisconsin School of Medicine and Public Health, University of Wisconsin-Madison, Madison, WI USA

**Keywords:** Shunt response, Cerebrospinal fluid, Idiopathic normal pressure hydrocephalus, Fluid biomarkers, Proteomics, Tandem mass tag

## Abstract

**Background:**

Idiopathic Normal pressure hydrocephalus (iNPH) is a form of adult hydrocephalus that is clinically characterized by progressive gait impairment, cognitive dysfunction, and urinary incontinence. The current standard method of treatment involves surgical installation of a CSF diversion shunt. However, only a fraction of patients shows an alleviation of symptoms from shunt surgery. Thus, the purpose of this prospective explorative proteomic study was to identify prognostic CSF biomarkers to predict shunt responsiveness in iNPH patients. Further, we evaluated the ability of the core Alzheimer’s disease (AD) CSF biomarkers phosphorylated (p)-tau, total (t)-tau, and amyloid-β 1–42 (Aβ_1–42_) to serve as predictors of shunt response.

**Methods:**

We conducted a tandem mass tag (TMT) proteomic analysis of lumbar CSF from 68 iNPH patients, sampled pre-shunt surgery. Tryptic digests of CSF samples were labelled with TMTpro reagents. The TMT multiplex samples were fractionated in 24 concatenated fractions by reversed-phase chromatography at basic pH and analysed by liquid chromatography coupled to mass spectrometry (LC–MS) on an Orbitrap Lumos mass spectrometer. The relative abundances of the identified proteins were correlated with (i) iNPH grading scale (iNPHGS) and (ii) gait speed change 1 year after surgery from baseline to identify predictors of shunt responsiveness.

**Results:**

We identified four CSF biomarker candidates which correlated most strongly with clinical improvement on the iNPHGS and were significantly changed in shunt-responsive compared to shunt-unresponsive iNPH patients 1 year post-surgery: FABP3 (*R* = − 0.46, log_2_(fold change (FC)) = − 0.25, *p* < 0.001), ANXA4 (*R* = 0.46, log_2_(FC) = 0.32, *p* < 0.001), MIF (*R* = -0.49, log_2_(FC) =  − 0.20, *p* < 0.001) and B3GAT2 (*R* = 0.54, log_2_(FC) = 0.20, *p* < 0.001). In addition, five biomarker candidates were selected based on their strong correlation with gait speed change 1 year after shunt installation: ITGB1 (*R* = − 0.48, *p* < 0.001), YWHAG (*R* = − 0.41, *p* < 0.01), OLFM2 (*R* = 0.39, *p* < 0.01), TGFBI (*R* = − 0.38, *p* < 0.01), and DSG2 (*R* = 0.37, *p* < 0.01).

Concentrations of the CSF AD core biomarkers did not differ significantly with shunt responsiveness.

**Conclusion:**

FABP3, MIF, ANXA4, B3GAT2, ITGB1, YWHAG, OLFM2, TGFBI and DSG2 in CSF are promising prognostic biomarker candidates to predict shunt responsiveness in iNPH patients.

**Supplementary Information:**

The online version contains supplementary material available at 10.1186/s12987-023-00440-5.

## Background

Idiopathic Normal pressure hydrocephalus (iNPH) is a relatively common form of adult hydrocephalus [1] featuring disturbed cerebrospinal fluid (CSF) homeostasis. It is clinically mainly characterized by progressive gait impairment, while cognitive dysfunction and urinary incontinence are also frequently present [2, 3]. To date, the only treatment of iNPH to have shown effectiveness involves installation of a CSF diversion shunt [4, 5]. During shunting, a catheter is surgically placed into the cerebral ventricle of the patient to divert the CSF flow to an extracerebral space, thereby leading to a reduction of pressure exerted on the brain. Although some studies suggest significant clinical benefit in the vast majority of iNPH patients [6, 7], only a fraction of iNPH patients appears to benefit clinically from the shunt procedure with a long-term alleviation of symptoms [8]. In practice, the prediction of long-term outcomes has been conducted alongside systematic diagnostic workup accompanied by prognostic tests such as the CSF tap test, albeit with limited success [9, 10]. Importantly, both the invasive nature of the shunt procedure and common occurrence of significant adverse effects [11] emphasize the need for more robust preoperative prediction tools for the treatment team.

While there are no widely established biomarkers to predict the treatment outcome, some fluid, mainly CSF, biomarkers have been proposed [12–14]: phosphorylated (p)-tau, total (t)-tau and amyloid-β 1–42 (Aβ_1–42_) are among the most intensively studied. High CSF p-tau and t-tau concentrations as well as a low CSF p-tau/Aβ_1–42_ ratio were found to be associated with unfavourable outcome after shunt surgery [13, 14]. These findings are consistent with the typical CSF core biomarker signature of Alzheimer’s disease (AD) [15], which has been shown to be frequently concomitant with iNPH [16]. Despite several studies suggesting an association of AD CSF biomarkers with shunt responsiveness [12, 17–19], other studies failed to reproduce similar results [20–23] casting doubt on the prognostic value of these, otherwise useful, diagnostic markers. Limitations of previous studies include a comparatively small number of shunt-nonresponsive patients and a limited clinical follow-up [14]. Comparability among studies is further hampered by differing inclusion criteria for shunt surgery as well as non-uniform clinical assessment scales for evaluating shunt responsiveness. Taken together, this underlines the need for a sufficiently large, long-term study investigating novel CSF biomarkers to predict shunt responsiveness in iNPH patients.

To this end, we conducted a tandem mass tag (TMT) proteomic analysis of preoperative lumbar CSF from 68 thoroughly phenotyped iNPH patients with objective and clinician-rated outcome measures 1 year after shunting to identify novel biomarkers to better predict shunt responsiveness. To be as applicable as possible to clinical practice, patients with preoperatively diagnosed neurodegenerative comorbidities prior referral were not excluded.

## Methods

### Kuopio NPH registry

The Kuopio NPH and AD Registry and Tissue bank included patients from Eastern Finnish population, referred to the KUH neurosurgical unit for suspected NPH [9]. The lenient inclusion criteria for the registry allows wide range of hydrocephalic conditions and comorbidities [9]: the patient must exhibit one to three symptoms possibly related to NPH (impaired gait, cognition, or urinary continence) together with enlarged brain ventricles (Evans’ index > 0.3) in computer tomography (CT) or magnetic resonance imaging (MRI) and no other explicit cause sufficient to alone explain observed findings and symptoms [9]. Preoperative comorbidities and conditions were recorded at baseline and patients underwent systematic differential diagnostic workup [9], followed by CSF tap test paired with gait evaluation [9]. Follow-up was conducted on all operated patients. The optimal shunt function was guaranteed by (1) valve adjustment, (2) brain imaging, (3) shunt valve tapping, (4) lumbar infusion test and (5) shunt revision if needed.

### Study population

The cohort consisted of 68 lumbar CSF samples (acquired during CSF tap test) from consecutive patients referred to KUH 2013–2021 with adult hydrocephalus with adequate CSF volume available: 68 possible or probable iNPH (1) (Table 1). Preoperative probabilities of iNPH are presented according to both international (2) and Japanese (3) criteria (Table 1). Thirteen patients with possible iNPH had neurodegenerative disease diagnosis made by a neurologist or geriatrician specialized in memory disorders prior shunting: 7 AD, 3 AD with vascular cognitive impairment, 2 Parkinson’s disease and one with frontotemporal dementia (C9ORF72). Information whether AD diagnoses were made according to the revised NINCDS-ADRDA criteria or IWG-2 criteria [24] could not be obtained.

**Table 1:** Cohort demographics

	iNPH		iNPH with neurodegenerative disease	
	Responsive (n = 28)	Unresponsive (n = 27)	Responsive (n = 7)	Unresponsive (n = 6)
Sex (male)	15/28 (53.6%)	16/27 (59.3%)	5/7 (71.4%)	4/6 (66.7%)
Age at shunting	72.5 [69.0, 78.0]	73.0 [70.0, 78.0]	81.0 [77.5, 85.0]	78.0 [75.5, 80.5]
Preoperative iNPH probability, Guidelines for Management of Idiopathic Normal Pressure Hydrocephalus, 3rd edition	Modified criteria^a^: 26 probable 2 possible	Modified criteria^a^: 25 probable 2 possible	Modified criteria^a^: 5 probable 2 possible	Modified criteria^a^: 6 probable 0 possible
Preoperative iNPH probability, Diagnostic classification of iNPH, International guidelines	Modified criteria^a^: 20 probable 8 possible	Modified criteria^a^: 24 probable 3 possible	Modified criteria^a^: 0 probable 7 possible	Modified criteria^a^: 0 probable 6 possible
Charlson-Age Comorbidity Index	5.00 [4.00, 6.00]	5.00 [4.00, 6.00]	6.00 [6.00,6.50]	5.00 [5.00,6.50]
Major secondary conditions^b^				
AD	0	0	5	2
PD	0	0	2	0
AD + VCI	0	0	0	3
FTD	0	0	0	1
Biopsy status				
Ab –, Tau –	20 (71.4%)	14 (51.9%)	1 (14.3%)	2 (33.3%)
Ab + , Tau –	7 (25.0%)	12 (44.4%)	4 (57.1%)	2 (33.3%)
Ab + , Tau +	1 (3.6%)	1 (3.7%)	3 (28.6%)	2 (33.3%)
CSF p-tau	34.3 [27.5, 40.7]	33.1 [27.0, 40.6]	36.0 [35.1, 37.0]	54.4 [26.2, 64.0]
CSF t-tau	153 [129, 218]	170 [111, 217]	203 [188, 267]	360 [228, 413]
CSF Aβ_1-42_	736 [628, 814]	671 [505, 815]	396 [372, 602]	465 [406, 625]
CSF p-tau/ Aβ_1-42_	0.047 [0.039, 0.062]	0.050 [0.037, 0.070]	0.093 [0.078, 0.097]	0.073 [0.050, 0.150]
INPHGS				
Baseline	6.50* [4.00, 8.25]	4.00 [3.00, 6.00]	7.00 [7.00,9.00]	6.50 [5.25,7.75]
3 months postop	4.00 [2.00, 6.00] (n = 27)	5.00 [3.00, 7.00] (n = 22)	6.00 [5.00, 7.00]	6.00 [6.00, 7.50]
12 months postop	3.00** [1.00, 5.25]	7.00 [4.00, 9.00]	6.00* [5.50, 6.00]	9.00 [8.25, 9.75]
point improvement from baseline to 3 months	2.00*** [1.00, 3.00] (n = 27)	0 [− 1.00, 1.00] (n = 22)	1.00 [0,3.00]	− 0.500 [− 1.00, 2.25]
point improvement from baseline to 12 months	2.00*** [1.00, 3.00]	− 1.00 [− 2.00, − 0.500]	2.00** [1.00, 3.00]	− 2.00 [− 2.00, − 1.25]
Gait speed (m/s)				
Baseline	0.548 [0.356, 0.865] (n = 27)	0.714 [0.476, 0.896]	0.696 [0.405, 0.861]	0.641 [0.436, 0.863]
Post-tap	0.784 [0.524, 0.983] (n = 27)	0.880 [0.578, 1.00] (n = 26)	0.746 [0.489, 0.899] (n = 6)	0.818 [0.656, 1.08]
3 months postop	0.902 [0.615, 1.25] (n = 26)	0.820 [0.510, 1.12]	0.870 [0.458, 1.07]	0.788 [0.621, 1.13]
12 months postop	0.909 [0.738, 1.29] (n = 24)	0.909 [0.659, 1.27] (n = 19)	0.884 [0.666, 1.02] (n = 6)	0.870 [0.781, 0.889] (n = 5)
speed improvement from baseline to 3 months	0.224* [0.139, 0.378] (n = 26)	0.137 [0.0114, 0.279]	0.225 [0.00291, 0.272]	0.234 [0.136, 0.314]
speed improvement from baseline to 12 months	0.296* [0.232, 0.461] (n = 24)	0.171 [− 0.0777, 0.370] (n = 19)	0.154 [− 0.0292, 0.355] (n = 6)	0.0697 [− 0.104, 0.274] (n = 5)
CERAD				
Baseline	64.0 [56.0, 71.0] (n = 25)	61.0 [53.0, 66.3] (n = 26)	59.5 [58.0, 61.0] (n = 6)	52.0 [45.0, 55.0] (n = 5)
3 months postop	67.0 [58.0, 73.0] (n = 23)	64.0 [62.0, 72.0] (n = 25)	55.5 [51.8, 63.0] (n = 6)	47.0 [43.0, 50.3]
12 months postop	69.0 [58.5, 76.5] (n = 26)	67.0 [55.0, 74.0] (n = 22)	59.5 [57.0, 68.8] (n = 6)	48.0 [44.0, 49.0] (n = 5)
MMSE				
Baseline	24.0 [21.0, 27.0] (n = 27)	25.0 [20.0, 26.0]	23.0 [18.0, 24.5]	19.5 [19.0, 22.3]
3 months postop	25.0 [23.0, 28.0] (n = 25)	25.0 [22.3, 26.8] (n = 26)	23.0 [20.0, 24.5]	20.0 [18.5, 22.3]
12 months postop	27.5* [24.0, 28.0] (n = 26)	24.5 [21.8, 26.3] (n = 24)	22.0 [18.5, 24.5]	16.0 [16.0, 22.0] (n = 5)

Demographic characteristics and follow-up variables of the iNPH cohort. Corresponding *p*-values were calculated employing chi-square goodness of fit test for categorical variables and Kruskal–Wallis test for continuous variables. Continuous variables are displayed as ‘median [Q1, Q3]’. In case of missing values, the respective number of observations is indicated. *p < 0.05, **p < 0.01, ***p < 0.001 compared to respective unresponsive group

^a^criteria regarding CSF opening pressure were removed, as it was measured only in patients going through infusion tests in this study population

^b^secondary condition as determined before shunting

### Outcome measures

#### iNPH grading scale

A modified Finnish version of the 12-point iNPH Grading Scale (iNPHGS) was used to assess severity of NPH related symptoms [9, 25]. INPHGS is a clinician-rated scale to separately estimate the severity of each of the triad symptoms with a scoring based on interviews with the patients or their caregivers and observations by the physician [9, 25]. Higher scores represent more severe symptoms [9, 25]. A minimum of one-point reduction in the iNPHGS has been considered a clinically observable improvement in the patient’s condition [9, 25, 26]. INPHGS was recorded at each time point (preoperatively, 3 and 12 months postoperatively) (Table 1), and we focused on the long-term (1 year) outcome.

#### Cognitive testing

The Consortium to Establish a Registry for Alzheimer’s Disease Neuropsychological Battery (CERAD-NB) [27] was used to measure cognitive impairment in study participants. The Finnish version of the CERAD-NB test battery includes nine subtests [28], one of which is Mini-Mental State examination (MMSE) [29]. CERAD total score can be calculated by summing up scores from the individual CERAD subtests (excluding MMSE), with lower scores indicating lower cognitive performance [30]. The CERAD-NB was conducted by CERAD-NB trained research nurse and recorded at each time point.

#### Gait speed

Gait evaluation was performed according to the Kuopio iNPH protocol [9]. In May 2017, there was an update regarding the previously described gait speed task [9]. For patients undergoing gait speed (meters per second, m/s) evaluation prior to the update, respective tasks included walking for 10 m, rotating, and returning to the starting point (20 m in total), repeated twice at each time point. For the rest of the study population, gait speed tasks included walking for 10 m, repeated thrice at each time point. After the update, a stopwatch accurate to two decimal places was used (instead of an accuracy of one second used prior). Gait speed was set to be 0 m/s for 4 patients who were unable to perform the gait speed test even with assistance. One patient who underwent gait evaluation had missing gait speed information at baseline.

### CSF collection and measurement of CSF AD core biomarkers

CSF samples (20–40 mL) were collected during the tap tests and collected in 14 mL polypropylene tubes, divided into aliquots of 250 µL, and immediately frozen at − 80 °C until analysis. CSF Aβ_1–42_, t-tau and p-tau were quantified at the UEF Biomarker Laboratory. Samples were analyzed either with ELISA assays (before 2020, assays Innotest β-amyloid (1–42), Innotest hTAU-Ag, and Innotest Phospho-Tau (181P), Fujirebio Europe, 30–33) or with automated immunoassays (since 2020, assays Elecsys β-Amyloid (1–42) CSF, Elecsys Total Tau CSF, and Elecsys Phospho-Tau (181P) CSF, Roche Diagnostics, 34–35). Due to measurement level differences between Innotest and Elecsys methods [36], the following conversions were performed to enable direct comparison of the results: Aβ_1–42_ Elecsys = (1.22 × Aβ_1-42_ Innotest) + 7.15, t-tau Elecsys = (0.475 × t-tau Innotest) + 66.0, p-tau Elecsys = (0.419 × p-tau Innotest)—3.807). These conversion factors had previously been established by measuring 100 CSF samples with Innotest and Elecsys assays.

### Brain biopsy

Three cylindrical frontal cortical biopsies (~ 2 mm in diameter and 3–10 mm in length) were acquired from each patient using disposable Temno Evolution^®^ TT146 biopsy needle at the site where the ventricular catheter would penetrate the brain (~ 3 cm from the midline and anterior to the coronal suture) [9]. Samples were stained using 6F3D and AT8 antibodies, evaluated by a neuropathologist, and graded semi-quantitatively for presence of Aβ plaques and tau tangles using light microscopy [9, 31].

### CSF sample preparation

CSF aliquots (25 µL) were reduced by the addition of 6.5 µL 24.2 mM Tris(2)-carboxyethylphosphine (TCEP) in 5% sodium deoxycholate (DOC), 0.5 M triethylammonium bicarbonate (TEAB), and subsequently heated at 55 °C for one hour. Following equilibration to room temperature, 1.6 µL of 200 mM iodoacetamide were added to the samples for carbamidomethylation. The samples were then incubated in the dark for 30 min. Trypsin (100 µg per vial; Promega) was dissolved in 500 µL resuspension buffer and 2.6 µg were added to each sample, followed by an overnight incubation at 37 °C. The next day, TMTpro reagents (TMT 18plex, Thermo Fisher, 5 mg) were equilibrated to room temperature, dissolved in 200 µL acetonitrile (ACN) and 10 µL were added to each sample. Samples were incubated at room temperature for one hour with constant agitation. The labelling reaction was then quenched by the addition of 3.2 µL 5% hydroxylamine solution and incubating for 30 min. Labelled samples were combined into corresponding TMT sets and diluted with 0.1% trifluoroacetic acid (TFA) to lower the ACN concentration to < 3%. DOC precipitation was performed by acidifying the pooled samples with hydrochloric acid (HCl). The precipitate was spun down at 4000 g for 15 min at 4 °C and the resulting supernatant was desalted by solid phase extraction (SPE) employing reversed-phase C_18_ cartridges (Sep-Pak C18 light) with a vacuum manifold. After washing of the cartridges with 1000 µL 0.1% TFA, 80% ACN and equilibration with 2 × 1000 µL 0.1% TFA, pooled samples were loaded onto the column. The column was washed twice with 1000 µL 0.1% TFA and peptides were eluted with 1000 µL 0.1% TFA, 80% ACN. Finally, the eluate was split into four aliquots of equal volume and lyophilized by vacuum centrifugation. Aliquots were stored at − 20 °C until subsequent fractionation.

### Offline high-pH reverse phase HPLC sample fractionation

One sample aliquot was dissolved in 22 µL 2.5 mM NH_4_OH and 2 µL were loaded on an UltiMate^™^ 3000 Nano LC system for offline high-pH HPLC fractionation. Separation was performed on an XBridge BEH C_18_ column (pore size: 130 Å, inner diameter: 4.6 mm). The following gradient was employed for peptide elution: Buffer B ranging from 1–45% over a 65 min gradient (flow rate 10 µL/min), Buffer C = 10% (Buffer A: H_2_O, Buffer B: 84% ACN, Buffer C: 25 mM NH_4_OH). Fractions were collected at 1 min time intervals circling over two rows in a 96-well microtiter plate, resulting in 24 concatenated fractions. The column was then cleaned at 90% B, 10% C for 10 min and subsequently equilibrated at 1% B, 10% C for 10 min. Fractions were dried by vacuum centrifugation and stored at − 20 °C until LC–MS analysis.

### Liquid chromatography-mass spectrometry (LC–MS)

Sample analysis was performed on a nano-LC (Ultimate RSLC Nano, Thermo Scientific) equipped with a C_18_ trap column (PepMap Acclaim 300 µm mm * 5 mm, Thermo Scientific) and C_18_ separation column (PepMap Acclaim 75 µm * 500 mm, Thermo Scientific), connected to an Orbitrap Fusion^™^ Lumos^™^ Tribrid^™^ mass spectrometer (Thermo Scientific), fitted with an Easy Spray Source and a high-field asymmetric waveform ion mobility spectrometry (FAIMS) unit for spatial ion separation. The following gradient was employed for peptide separation: 5 min, 4% B; 6 min, 10% B; 74 min, 40% B; 75 min, 100% B (loading buffer: 0.05% TFA, 0.1% bovine serum albumin; Buffer A: 0.1% FA; Buffer B: 84% ACN, 0.1% FA). The mass spectrometer was operated in the positive ion mode. Alternating MS/MS cycles were performed (cycle time = 1.5 s) at compensation voltages (CV) of CV = − 50 V and CV = − 70 V, respectively. First, a full Orbitrap MS scan was recorded (R = 120 k, AGC target = 100%, max injection time = 50 ms), followed by data dependent Orbitrap MS/MS scans (isolation window = 0.7 m/z, activation type = HCD, R = 50 k, AGC target = 200%, max. injection time = 120 ms).Data processing and normalization.

Proteome Discoverer Version 2.5.0.400 (Thermo Scientific) was used for data processing. Peak integration for reporter ion quantification was performed with the integration method of most confident centroid (integration tolerance = 20 ppm). Peptides were identified using Sequest^HT^ search engine with UniProtKB Swiss-Prot (TaxID = 9606, Homo sapiens) set as database. The search parameters included precursor Δm tolerance = 5 ppm, fragment Δm tolerance = 0.02 Da, missed cleavages = 2, min. peptide length = 6, fixed modifications = carbamidomethyl, TMTpro (peptide N-terminus, K residues). Percolator was used for peptide scoring with an identification threshold of 1% false discovery rate (FDR). For quantification, peptide groups were considered based on their uniqueness (unique peptides) and in accordance with the principle of parsimony (razor peptides). Missing values were not imputed.

Scaled protein ratios were obtained by dividing each protein measurement by its corresponding measurement in the global internal standard channel (TMT channel 135N). Data normalization was then performed by dividing each protein ratio by the respective sample median. Proteins with more than 50% of missing values across all study participants were excluded from the analysis.

### Patient stratification and identification of biomarker candidates based on iNPHGS and gait velocity

A patient was considered as shunt responsive if a reduction by one or more points in the iNPHGS could be observed 1 year post-shunting. Otherwise, the patient was classified as unresponsive. To identify biomarker candidates of shunt responsiveness based on the iNPHGS, we pursued two approaches: (i) stratifying iNPH patients into a shunt-responsive and unresponsive group 1 year post-shunting and determining which proteins differed significantly in abundance between the two groups and (ii) correlating the respective protein abundances with clinical improvement on the iNPHGS 1 year after shunt placement. Correlation analysis circumvents the issue of having to define a cut-off for shunt responsiveness as is done in approach (i), which relies on group stratification and may introduce bias. By utilizing both approaches, analytically important parameters such as protein fold change (FC) can be acquired with approach (i) while approach (ii) provides additional confidence in the validity of the results. The identification of biomarker candidates based on gait velocity was performed by correlating changes in gait speed 1 year post-shunting with respective protein abundances. Patients were not stratified into shunt responsive and unresponsive based on gait speed since no validated minimal clinically important difference (MCID) for gait speed has been established to date.

### Statistical analysis

All statistical analyses were performed with R version 4.1.2. For analysis of covariance (ANCOVA), the data was log_2_-transformed to satisfy the requirement of a normal distribution. Both age and sex were included as covariates. Spearman’s rank-order correlation was used to measure the monotonic relationship between two variables. Statistically significant differences in the demographics characteristics of the cohort were evaluated with chi-square goodness of fit test and Kruskal–Wallis test for categorical and continuous variables, respectively. To assess significant differences in protein abundance across biopsy status groups, ANOVA was employed. *P*-values were adjusted with the Benjamini–Hochberg procedure (false discovery rate adjustment) wherever specified. One-year changes from baseline in the parameters iNPHGS, gait speed, MMSE and CERAD were calculated in such a fashion that a positive change value signified clinical improvement.

## Results

### Cohort demographics and AD CSF core biomarkers levels as predictors of shunt responsiveness in iNPH patients

Our study cohort included a total of 68 iNPH patients; subdivided into iNPH patients without comorbid neurodegenerative condition (n = 55), and iNPH patients with a clinical diagnosis of neurodegenerative disease prior to shunting (n = 13) (Table 1). All patients underwent shunt surgery, and their clinical symptoms were evaluated at baseline, 3 months post-shunting as well as 1 year after shunt placement according to (i) the iNPHGS and (ii) objective outcome measurements including gait speed, CERAD and MMSE scores. Patient stratification was performed based on shunt responsiveness as assessed via changes in the iNPHGS 1 year post-shunting. Thus, in this study, we focused on the long-term (1 year) clinical benefits from shunting as opposed to short-term (3 months) effects. The iNPHGS was chosen for patient stratification as it encompasses the entire triad of symptoms and provides a validated minimal clinically important difference (MCID) [25].

28/55 (51%) iNPH patients and 7/13 (54%) iNPH patients with comorbid neurodegenerative condition had iNPHGS MCID 1 year after shunting (Table 1). Within the group of iNPH patients without comorbid neurodegenerative condition, both shunt-responsive and unresponsive patients improved in gait speed 3 months as well as 1 year postoperatively. However, the improvement was significantly greater in shunt-responsive compared to unresponsive patients (p < 0.05). iNPH patients with a neurodegenerative disease likewise showed an improvement in gait speed at both clinical follow-up time points, though the change in speed did not differ significantly between both groups (Table 1). Cognitive profiles were different across iNPH patients with and without clinical signs of comorbid neurodegenerative condition at the time of iNPH diagnosis: patients with a neurodegenerative disease displayed consistently lower CERAD and MMSE scores (Table 1).

Shunting permits the surgeon to collect brain cortex samples from the patient, which can be pathologically examined for the presence of Aβ-plaques (Aβ–/ +) or tau neurofibrillary tangles (tau–/ +), constituting to the two key neuropathologies in AD. Interestingly, despite no neurodegenerative disease having been clinically diagnosed, approximately 30% of the shunt-responsive (n = 28) and ca. 50% of the shunt-unresponsive group (n = 27) of iNPH patients exhibited plaque and/or neurofibrillary tangle positivity upon biopsy (Table 1).

Due to conflicting study results in the literature, we tested the ability of the AD CSF core biomarkers Aβ_1–42_, p-tau, t-tau, and the p-tau/Aβ_1–42_ ratio, measured at baseline, to predict shunt responsiveness in iNPH patients. No significant difference between shunt-responsive and shunt-unresponsive iNPH patients could be found for any of the biomarkers, suggesting that they hold limited predictive value.

### Selection of biomarker candidates to predict shunt responsiveness based on the iNPHGS

Employing TMT proteomics, 2795 proteins were identified in the lumbar CSF of patients sampled pre-surgery, 1860 of which could be quantified in > 50% of iNPH patients. To identify biomarker candidates of shunt responsiveness based on the iNPHGS, we (i) stratified iNPH patients into a shunt-responsive and unresponsive group 1 year post-shunting and determined which proteins differed significantly in abundance between the two groups (Additional file 1: Fig S1) and (ii) correlated the respective protein abundances with clinical improvement on the iNPHGS 1 year after shunt placement. To be as applicable as possible to clinical practice, patients with and without preoperatively diagnosed neurodegenerative comorbidities were combined for the analysis (total: n = 68).

Table 2 displays the top ten biomarker candidates obtained through correlation analysis while Table 3 shows the top ten biomarker candidates identified with ANCOVA, considering age and sex as covariates. Notably, in both approaches almost all false discovery rate (FDR)-adjusted *p*-values are larger *p* > 0.05, potentially owing to the low number of observations in each group (n = 35, n = 33), high number of variables (n = 1860) and moderate fold-change.

**Table 2:** Top ten list of biomarker candidates correlating most strongly with iNPHGS change 1 year post-shunting

Accession	Gene symbol	Spearman correlation coefficient *R*	*p*-value	FDR-adjusted *p*-value
P14174	MIF	− 0.49	< 0.001	0.046
P05413	FABP3	− 0.46	< 0.001	0.067
P16152	CBR1	− 0.44	< 0.001	0.080
P09525	ANXA4	0.46	< 0.001	0.080
Q9NPZ5	B3GAT2	0.54	< 0.001	0.080
Q9UJ14	GGT7	− 0.46	< 0.001	0.080
A0A0B4J1V7	IGHV7-81	0.42	< 0.001	0.080
P37837	TALDO1	− 0.42	< 0.001	0.080
P06744	GPI	− 0.42	< 0.001	0.080
P06733	ENO1	− 0.42	< 0.001	0.080

List of top ten biomarker candidates to predict shunt responsiveness in iNPH patients 1 year post-shunting identified via Spearman rank-order correlation. Protein abundances were correlated with the patient’s corresponding improvement on the iNPHGS 1 year after shunt installation. *P*-values were adjusted using Benjamini–Hochberg correction. Biomarker candidates overlapping with the top ten list obtained through ANCOVA analysis are marked in blue

**Table 3:** Top ten list of biomarker candidates most significantly changed between shunt-responsive and shunt-unresponsive iNPH patients

Accession	Gene Symbol	*p*-value	FDR-adjusted *p*-value	log_2_(fold change)
P05413	FABP3	< 0.001	0.025	− 0.25
P62328	TMSB4X	< 0.001	0.065	− 0.23
O43491	EPB41L2	< 0.001	0.065	0.27
P62258	YWHAE	< 0.001	0.065	− 0.18
P14174	MIF	< 0.001	0.065	− 0.20
Q9HDC9	APMAP	< 0.001	0.078	0.50
Q9NPZ5	B3GAT2	< 0.001	0.078	0.20
P54652	HSPA2	< 0.001	0.078	− 0.21
P02655	APOC2	< 0.001	0.078	0.71
P09525	ANXA4	< 0.001	0.078	0.32

List of top ten biomarker candidates to predict shunt responsiveness in iNPH patients 1 year post-shunting identified via analysis of covariance (ANCOVA), employing age and sex as covariates. Patients were grouped into shunt-responsive (n = 35) and unresponsive (n = 33) according to their clinical improvement on the iNPHGS 1 year after shunt installation. *P*-values were adjusted using Benjamini–Hochberg correction. Biomarker candidates overlapping with the top ten list obtained through correlation analysis are marked in blue

Four proteins were identified to be among the top ten ranking biomarker candidates by both approaches: Fatty acid-binding protein, heart (FABP3), Macrophage migration inhibitory factor (MIF), Annexin A4 (ANXA4) and glyceraldehyde-3-phosphate dehydrogenase (B3GAT2). Thus, we propose these four proteins as the top biomarker candidates to predict shunt responsiveness in iNPH patients 1 year after shunting based on the iNPHGS.

To evaluate whether the association of the selected markers with shunt-responsiveness was potentially driven by neurodegenerative processes, we repeated both correlation and ANCOVA analyses, excluding iNPH patients with comorbid neurodegenerative condition prior to shunting (n = 55). After exclusion of this patient group, all four proteins remained significantly associated with shunt-responsiveness showing negligible changes in correlation coefficients (Additional file 3: Table S1) and log_2_-FC (Additional file 3: Table S2). In addition, we assessed protein abundances of the top four biomarker candidates across the biopsy status groups Aβ^−^/tau^−^, Aβ^+^/tau^−^, and Aβ^+^/tau^+^ (Additional file 2: Fig S2A–D). FABP3 was the only biomarker candidate for which a significant difference between biopsy status groups could be determined (*p* < 0.01), suggesting that its abundance increases as neurodegeneration progresses.

### Selection of biomarker candidates to predict shunt responsiveness based on the objective outcome measurement gait speed

The iNPHGS encompasses the symptomology of iNPH well, incorporating all three key symptoms gait disturbance, cognitive impairment, and urinary incontinence into a single scale. However, as a clinician-rated scale, it remains subjective and therefore potentially inconsistent. Thus, besides selecting biomarkers based on iNPHGS only, we also assessed the correlation of protein abundances with change in the objectively quantifiable measurement gait speed, the hallmark feature of patients with iNPH [2]. A MCID for gait speed change has been suggested for adults with pathology [32], however, it has not been validated for iNPH specifically. Consequently, we solely focused on a correlation analysis to identify biomarkers of shunt-responsiveness based on gait speed change. Again, both iNPH patients with and without comorbid neurodegenerative disease with available gait speed data (n = 53) were included in the analysis.

Table 4 displays the top five biomarker candidates based on their correlation with change in gait speed 1 year post-shunting from baseline.

**Table 4:** Top five list of biomarker candidates correlating most strongly with gait speed change 1 year post-shunting

Accession	Gene symbol	Spearman correlation coefficient *R*	*p*-value	FDR-adjusted *p*-value
P05556	ITGB1	− 0.48	< 0.001	0.755
P61981	YWHAG	− 0.41	< 0.01	0.984
O95897	OLFM2	0.39	< 0.01	0.984
Q15582	TGFBI	− 0.38	< 0.01	0.984
Q14126	DSG2	0.37	< 0.01	0.984

List of top five biomarker candidates to predict shunt responsiveness in iNPH patients 1 year post-shunting identified via Spearman rank-order correlation. Protein abundances were correlated with the patient’s corresponding change in gait speed 1 year after shunt installation. *P*-values were adjusted using Benjamini–Hochberg correction

Integrin beta-1 (ITGB1), 14-3-3 protein gamma (YWAG), Noelin-2 (OLFM2), Transforming growth factor-beta-induced protein ig-h3 (TGFBI), and Desmoglein-2 (DSG2) correlated most strongly with gait speed change 1 year post-surgery. To evaluate whether the correlation was mainly driven by patients with a comorbid neurodegenerative condition, we excluded this patient group and repeated the analysis (Additional file 3: Table S1). Both significance values and correlation coefficients were only slightly altered following the exclusion of iNPH patients with a clinically diagnosed neurodegenerative disease. Further, we investigated whether the protein abundances changed across different biopsy status groups (Additional file 2: Fig S2E–I). YWHAG differed significantly (*p* < 0.05) in abundance across biopsy status.

Notably, none of the top ten biomarkers determined via iNPHGS overlapped with the top five biomarkers identified via gait speed change, most likely because changes on the iNPHGS only moderately correlated with changes in gait speed (Additional file 3: Table S3).

### Correlations of selected biomarker candidates with different clinical parameters

Having identified potential biomarker candidates for long-term shunt-response via iNPHGS and gait speed measurements, we performed correlation analyses for all nine proteins with changes from baseline in the (i) iNPHGS, (ii) gait speed, (iii) CERAD score as well as (iv) MMSE score 1 year after shunting.

**Fig. 1:**
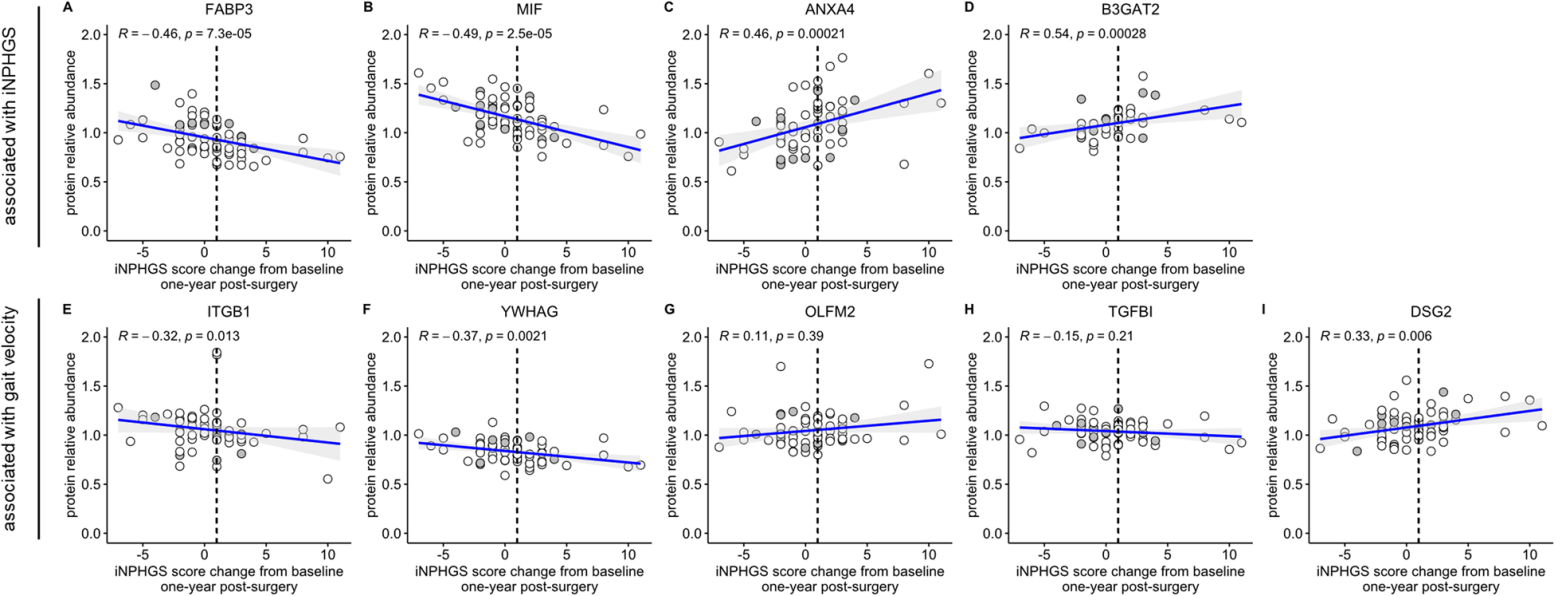
Correlation of top nine biomarker candidates with iNPHGS change. Spearman rank-order correlations of CSF FABP3 (**A**), MIF (**B**), ANXA4 (**C**), B3GAT2 (**D**), ITGB1 (**E**), YWHAG (**F**), OLFM2 (**G**), TGFBI (**H**), and DSG2 (**I**) with clinical improvement on the iNPH grading scale (iNPHGS) 1 year post-surgery. Clinical improvement was calculated by subtracting the iNPHGS measurement 1 year after shunt installation from the iNPHGS score baseline measurement so that a score change of >  = 1 (vertical dashed line) corresponded to a clinically significant shunt response. Points colored in gray represent measurements stemming from iNPH patients with a concomitant neurodegenerative disease. Displayed *p*-values were not FDR-corrected. The gray shading around the fitted line indicates the 95% confidence interval of the fit

Low concentrations of CSF FABP3 (*R* = − 0.46, *p* = 7.3e-05), MIF (*R* = − 0.49, *p* = 2.5e-05), ITGB1 (*R* = − 0.32, *p* = 0.013), and YWHAG (*R* = − 0.37, *p* = 2.1e-03) were found to be significantly associated with favourable outcome after shunt-surgery on the iNPHGS (Fig. 1A, B, E, F). In contrast, high concentrations of ANXA4 (*R* = 0.46, *p* = 2.1e-04), B3GAT2 (*R* = 0.54, *p* = 2.8e-04), and DSG2 (*R* = 0.33, *p* = 6e-03) significantly correlated with clinical improvement measured on the iNPHGS (Fig. 1C, D, I). OLFM2 (*R* = 0.11, *p* = 0.39) and TGFBI (*R* = − 0.15, *p* = 0.21) were not significantly associated with changes on the iNPHGS 1 year post-shunting (Fig. 1G, H).

**Fig. 2:**
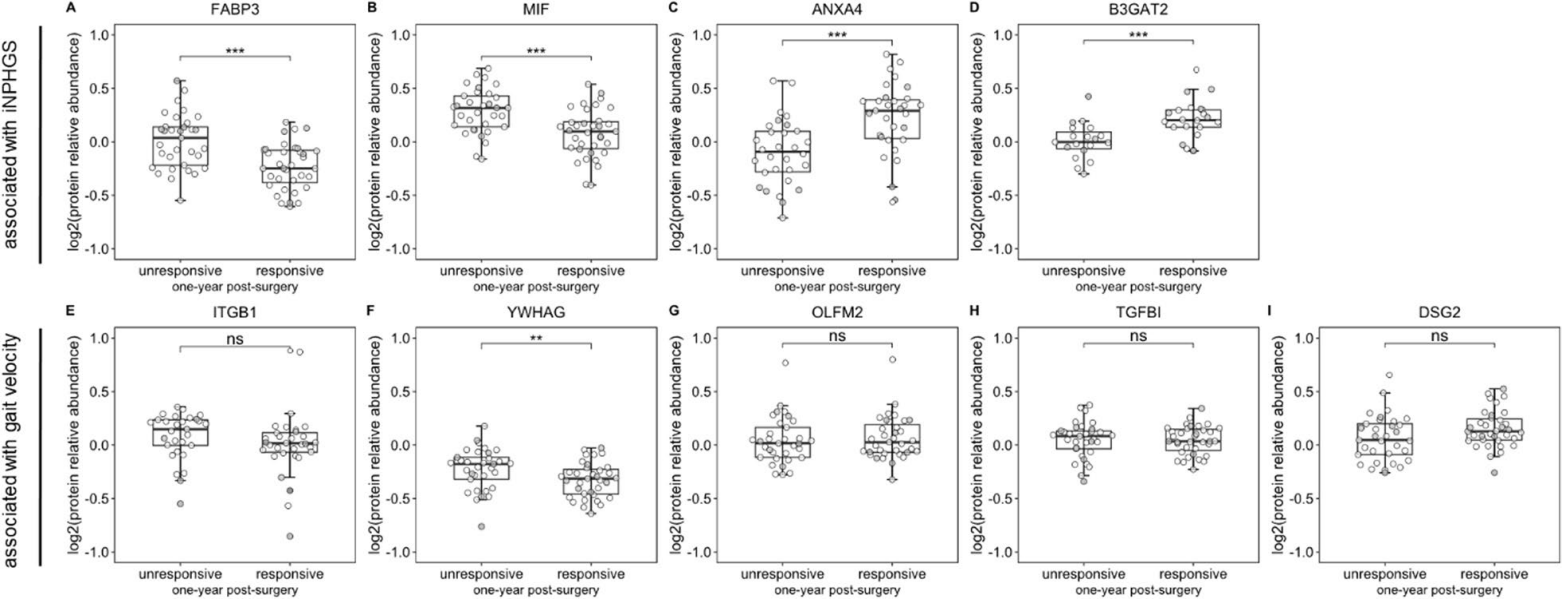
Boxplots of protein abundances of the top nine biomarkers across shunt-responsive and shunt-unresponsive iNPH patients. CSF protein abundances of FABP3 (**A**), MIF (**B**), ANXA4 (**C**), B3GAT2 (**D**), ITGB1 (**E**), YWHAG (**F**), OLFM2 (**G**), TGFBI (**H**), and DSG2 (**I**) in shunt-responsive and shunt-unresponsive iNPH patients 1 year after shunt installation. Points colored in gray represent measurements stemming from iNPH patients with a concomitant neurodegenerative disease. *P*-values (not FDR-corrected) were obtained by performing analysis of covariance (ANCOVA), using age and sex as covariates. *p < 0.05, **p < 0.01, ***p < 0.001, ns = not significant

Comparing biomarker levels between shunt-responsive and unresponsive patients stratified based on iNPHGS change 1 year after shunt installation, only YWHAG concentrations differed significantly (*p* < 0.01) among both groups (Fig. 2F) in addition to the proteins that had already been selected based on significant differences in this metric (FABP3, MIF, ANXA4, B3GAT2; Fig. 2 A-D).

**Fig. 3:**
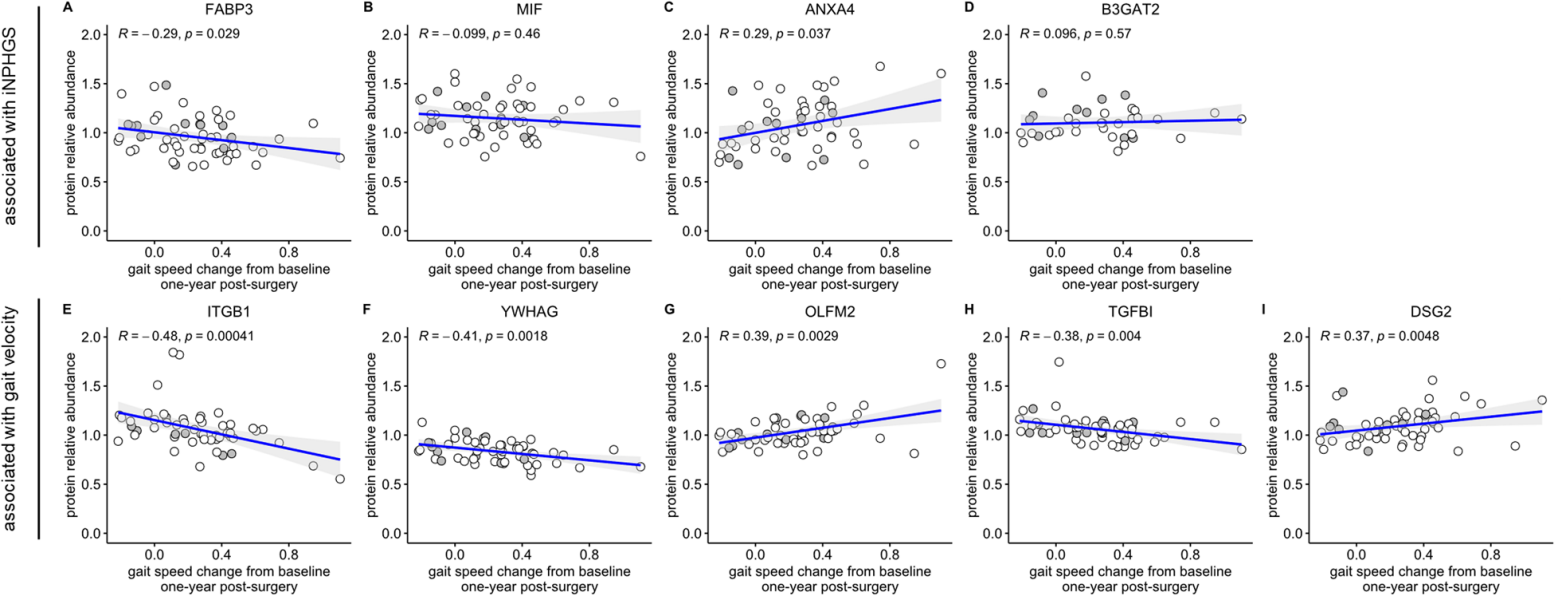
Correlation of top nine biomarker candidates with gait speed change. Spearman rank-order correlations of CSF FABP3 (**A**), MIF (**B**), ANXA4 (**C**), B3GAT2 (**D**), ITGB1 (**E**), YWHAG (**F**), OLFM2 (**G**), TGFBI (**H**), and DSG2 (**I**) with gait speed change 1 year post-surgery. Gait speed change was calculated by subtracting the gait speed measurement 1 year after shunt installation from the gait speed baseline measurement. Points colored in gray represent measurements stemming from iNPH patients with a concomitant neurodegenerative disease. Displayed *p*-values were not FDR-corrected. The gray shading around the fitted line indicates the 95% confidence interval of the fit

In addition to the five proteins identified via a significant correlation with gait speed change 1 year post-surgery (ITGB1, *R* = − 0.48, *p* = 4.1e-04; YWHAG, *R* = − 0.41, *p* = 1.8e-03; OLFM2, *R* = 0.39, *p* = 2.9e-03; TGFBI, *R* = − 0.38, *p* = 4.0e-03; and DSG2, *R* = 0.37, *p* = 4.8e-03), only FABP3 (*R* = − 0.29, *p* = 0.029) and ANXA4 (*R* = 0.29, *p* = 0.037) were significantly associated with changes in gait speed 1 year after shunt placement (Fig. 3A, C, E–I). Both MIF and B3GAT2 showed no significant relationship with alterations in gait speed (Fig. 3B, D). For each protein, the direction of the association remained unchanged compared to the iNPHGS correlation analysis.

**Fig. 4:**
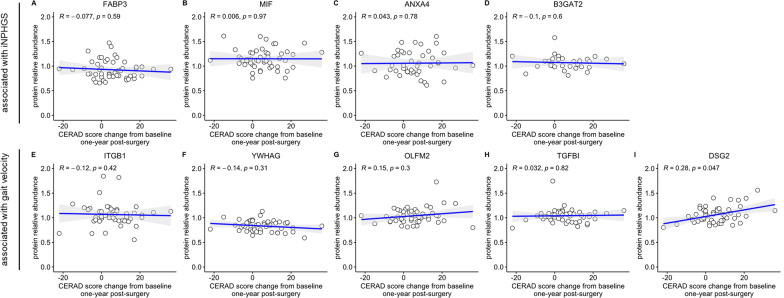
Correlation of top nine biomarker candidates with CERAD score change. Spearman rank-order correlations of CSF FABP3 (**A**), MIF (**B**), ANXA4 (**C**), B3GAT2 (**D**), ITGB1 (**E**), YWHAG (**F**), OLFM2 (**G**), TGFBI (**H**), and DSG2 (**I**) with Consortium to Establish a Registry for Alzheimer’s disease (CERAD) score change 1 year post-surgery. CERAD score change was calculated by subtracting the respective baseline measurement from the measurement obtained 1 year after shunting. Displayed *p*-values were not FDR-corrected. The gray shading around the fitted line indicates the 95% confidence interval of the fit

**Fig. 5:**
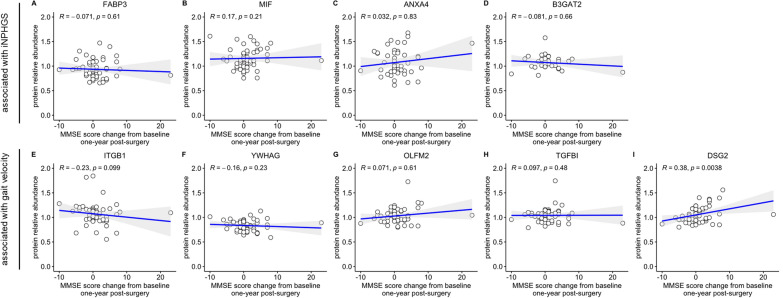
Correlation of top nine biomarker candidates with MMSE score change. Spearman rank-order correlations of CSF FABP3 (**A**), MIF (**B**), ANXA4 (**C**), B3GAT2 (**D**), ITGB1 (**E**), YWHAG (**F**), OLFM2 (**G**), TGFBI (**H**), and DSG2 (**I**) with mini mental state examination (MMSE) score change 1 year post-surgery. MMSE score change was calculated by subtracting the respective baseline measurement from the measurement obtained 1 year after shunting. Displayed *p*-values were not FDR-corrected. The gray shading around the fitted line indicates the 95% confidence interval of the fit

To determine the association of the top nine biomarker candidates with changes in cognitive parameters, correlation analyses were performed on iNPH patients without comorbid neurodegenerative conditions (n = 55) only. The progression of a diagnosed neurodegenerative disease such as AD is expected to severely affect a patient’s cognitive performance, potentially masking and confounding improvements in cognition stemming from shunting. Correlating the abundance of the top nine biomarker candidates with changes in CERAD and MMSE score, DSG2 emerged as the only protein to significantly correlate with both changes in CERAD (*R* = 0.28, *p* = 0.047) and MMSE score (*R* = 0.38, *p* = 3.8e-03) 1 year post-shunting (Figs. 3, 4I). Notably, none of the other biomarker candidates were significantly associated with either CERAD or MMSE score changes (Figs. 4, 5A–H).

**Fig. 6:**
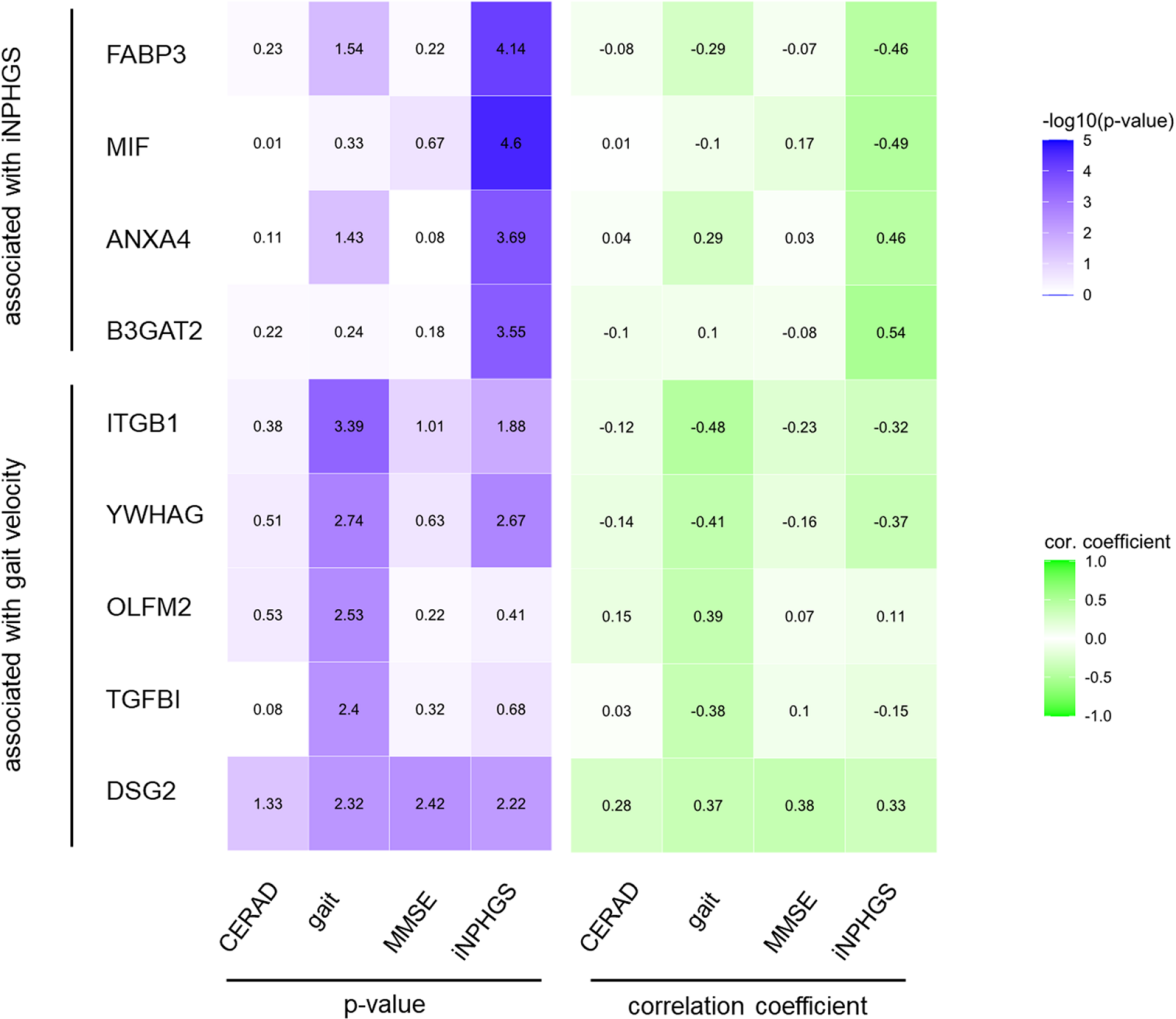
Heatmap of the top nine biomarker candidates summarizing analyses parameters. Heatmap of the top nine biomarker candidates’ significance values and correlation coefficients of the corresponding correlation analyses with CERAD score, gait speed, MMSE score and iNPHGS change 1 year after shunt installation. Displayed *p*-values were not FDR-corrected

Figure 6 summarizes the significance values and correlation coefficients of all corresponding correlation analyses in a heatmap. Integrating the results, DSG2 is the only protein to significantly correlate with all investigated parameters: changes in the iNPHGS, gait speed and cognition (CERAD and MMSE). Further, four out of the nine proposed biomarkers (FABP3, ANXA4, ITGB1 and YWHAG) were significantly associated with both iNPHGS and gait speed changes.

### Biomarker performance of selected biomarkers

We tested the ability of the biomarker candidates selected based on (i) iNPHGS change and of those chosen based on (ii) gait speed change to predict shunt responsiveness in iNPH patients 1 year post-surgery by performing a receiver operator characteristics (ROC) curve analysis. Patients were stratified into shunt-responsive and unresponsive according to the iNPHGS MCID criterion.

**Fig. 7:**
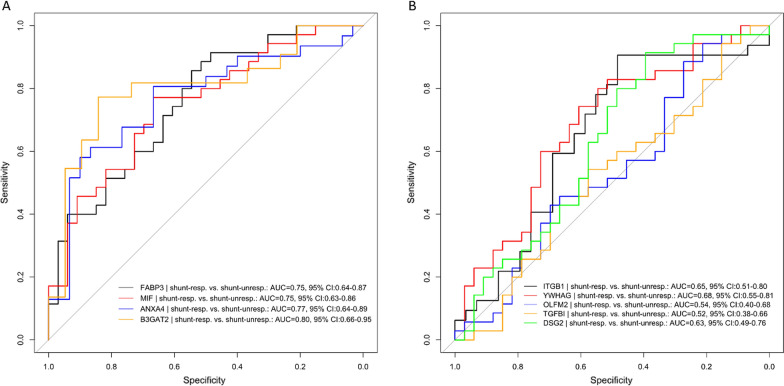
ROC curves for the top nine biomarker candidates discriminating shunt-responsive from shunt-unresponsive iNPH patients. Receiver operating characteristic (ROC) curves of (**A**) biomarker candidates chosen based on iNPHGS score change and (**B**) biomarker candidates selected based on gait speed change, calculated for shunt-responsive iNPH patients (n = 35) vs. shunt-unresponsive iNPH patients (n = 33) 1 year post-shunting. *AUC* Area under the ROC curve, *CI* Confidence interval

All biomarker candidates selected based on iNPHGS change displayed moderately high discriminatory ability to distinguish shunt-responsive from shunt-unresponsive patients 1 year after shunt installation. B3GAT2 discriminated shunt-responsive from shunt-unresponsive patients with an area under the ROC curve (AUC) of 0.80, closely followed by ANXA4 (AUC = 0.77), FABP3 (AUC = 0.75) and MIF (AUC = 0.75) (Fig. 7A). Because of the model’s bias, biomarker candidates chosen based on gait speed change performed considerably worse than their iNPHGS counterparts. YWHAG showed the best performance with an AUC of 0.68, while ITGB1 and DSG2 discriminated between both groups with an AUC of 0.65 and 0.63, respectively. OLFM2 (AUC = 0.54) and TGFBI (AUC = 0.52) did not perform better than a completely random classifier.

## Discussion

We herein present the first TMT proteomics study investigating novel CSF biomarkers to predict shunt responsiveness in iNPH patients. To the best of our knowledge, this is the largest proteomics study to identify markers of shunt responsiveness for iNPH patients to date. We propose a list of nine CSF biomarker candidates with the potential to predict 1 year shunt responsiveness in iNPH patients based on (i) the iNPHGS and (ii) the objective outcome measurement gait speed. The utilization of two different clinical parameters to identify biomarker candidates increases the probability of discovering proteins truly associated with shunt responsiveness.

FABP3, ANXA4, MIF and B3GAT2 emerged as the most promising biomarker candidates identified based on iNPHGS change while ITGB1, YWHAG, OLFM2, TGFBI and DSG2 could be determined as top biomarkers via their strong association with gait speed change 1 year post-shunting. Notably, there was no overlap among both lists of top biomarker candidates. However, out of all nine biomarker candidates, FABP3, ANXA4, ITGB1, YWHAG and DSG2 significantly correlated with both iNPHGS and gait speed change increasing the confidence in their association with long-term shunt response. Importantly, DSG2 also significantly correlated with changes in the cognitive parameters CERAD and MMSE rendering it the only protein out of all proposed biomarkers to be related to all clinical outcome measurements investigated in this study. Taken together, DSG2, FABP3, ANXA4, YWHAG and ITGB1 appear to be most strongly associated with long-term shunt response as they showed a significant correlation with at least 2 out of all 4 evaluated outcome measurements. The lack of overlap among both lists as well as the absence of a consistently significant correlation for all markers across outcome measurements is most likely rooted in the properties of the iNPHGS as comparatively weak outcome measurement [1, 10]. While the iNPHGS shows a moderate correlation with all objective outcome measurements, it is, as relatively crude categorical scale, insensitive to smaller clinical changes and does not reveal which symptom motivated a score change. In the present study, some patients exhibited relatively mild symptoms which may lead to a floor effect i.e., iNPHGS as outcome measurement may be too insensitive to optimally quantify shunt responsiveness and stratify patients. We believe that shunt failure, which could be erroneously interpreted as shunt unresponsive, is of negligible concern in the present cohort since optimal shunt function was ensured using a rigorous protocol. To assess each biomarker candidates’ capability to discriminate shunt-responsive from shunt-unresponsive patients, iNPH patients were stratified into respective groups based on iNPHGS. Consequently, biomarkers selected based on the iNPHGS metric performed considerably better in predicting outcome after shunting than the proteins identified based on gait speed change. Calculating ROC-curves for all candidates, B3GAT2 exhibited the highest discriminatory ability to predict shunt response with an AUC of 0.80.

To investigate a potential role of the proposed markers in iNPH or related conditions, we surveyed relevant literature in the corresponding scientific fields. Interestingly, most biomarker candidates related to either (i) brain injury, (ii) AD and/or (iii) cell adhesion. For instance, both elevated MIF and FABP3 have been previously shown to be linked to worse clinical outcome after subarachnoid hemorrhage and brain injury [33, 34]. Furthermore, a recent study demonstrated that FABP3 is an early outcome predictor in patients with traumatic brain injury [35]. Possibly, high levels of FABP3 and MIF are indicators of a severely injured brain thus making it more unlikely for such patients to benefit from shunting and for cerebral damage to revert significantly. On the other hand, FABP3 but also YWHAG and DSG2 appear to be associated with AD, which is frequently observed in iNPH patients. Several studies showed that both FABP3 and YWHAG were significantly increased in the CSF of AD patients compared to controls [36, 37], while DSG2 has been discovered as genetic risk factor for AD [38]. In the present study, we observed that both FABP3 and YWHAG concentrations appeared to increase with Aβ/tau positivity, further supporting a potential involvement in AD-related processes. The implication this bears on their suitability as shunt outcome biomarker, however, remains unclear. Finally, the biomarker candidates DSG2, OLFM2, B3GAT2, TGFBI and ITGB1 are all involved in cell–cell adhesion [39–44], more specifically TGFBI and ITGB1 have been suggested to play a role in blood brain barrier (BBB) permeability [45]. As the BBB and glymphatic system are both anatomically and functionally interconnected [46, 47] one might speculate that TGFBI and ITGB1 reflect glymphatic dysfunction, a recently discovered feature of iNPH [48, 49]. ANXA4, a Ca^2+^-dependent membrane-binding protein modulating membrane permeability has been linked to many cancer types [50], however, its association with iNPH remains elusive.

In accordance with previous studies [20–23], the AD CSF core biomarkers Aβ_1-42_, p-tau, t-tau, and the p-tau/Aβ_1-42_ ratio did not prove to be useful in predicting CSF shunting outcome. However, a notable, albeit insignificant difference in the core biomarkers among shunt-responsive and unresponsive patients could be observed. Potentially, these differences may become significant with an increased cohort size and power.

The strengths of this study include its large number of shunt-unresponsive patients compared to previous studies [12, 51], a very well-characterized cohort with extensive long-term follow-up clinical data as well as the utilization of two different clinical parameters, iNPHGS and gait speed, to identify markers of shunt-responsiveness. In addition, by employing TMT proteomics, we were able to evaluate the potential of thousands of CSF proteins to serve as shunt responsiveness predictors. On the other hand, it is noteworthy to mention that biomarker discovery for shunt responsiveness in iNPH patients is associated with several challenges, most of which are inherent to the disease and clinical assessment thereof [52]. First and foremost, the lack of a diagnostic gold standard impedes biomarker research as it negatively affects comparability among studies as well as prospective validation studies. In addition, differing clinical evaluation scales of iNPH complicate research work further. This study thus emphasizes the need for more robust and quantitative outcome measurements in the field of iNPH.

Future work should be directed at validating the proposed biomarkers in another cohort of iNPH patients with more precise CSF measurements via e.g., immunoassays or targeted MS assays. The complex nature of iNPH may require a panel of several biomarkers to reliably predict shunt responsiveness. We hope that our list of potential biomarker candidates can aid future research and ultimately result in a fluid biomarker to be implemented in the clinic as predictor of shunt-responsiveness in iNPH patients.

## Conclusion

We present a list of CSF biomarker candidates to predict shunt responsiveness in iNPH patients 1 year after shunt installation. The proteins FABP3, MIF, ANXA4, B3GAT2, ITGB1, YWHAG, OLFM2, TGFBI and DSG2 were found to be the top predictors of clinical outcome post-shunting in the present cohort. Further, the CSF AD core biomarkers p-tau, t-tau and Aβ_1-42_ did not differ significantly among shunt-responsive and unresponsive patients suggesting that they hold limited predictive value.

## Supplementary Information

**Additional file 1: Figure S1.** Volcano plot comparing CSF protein abundances of shunt-responsive (n=35) and shunt-unresponsive iNPH patients (n=33) one-year post-shunting. Log_2_-fold change (FC) cut-off: 0.1; *p*-value cut-off: 0.05. The *p*-values were not FDR-corrected.

**Additional file 2: Figure S2.** Log_2_-transformed protein abundance of the biomarker candidates FABP3 (A), MIF (B), ANXA4 (C) and B3GAT2 (D), ITGB1 (E), YWHAG (F), OLFM2 (G), TGFBI (H), and DSG (I) across the biopsy status groups Aβ^-^/tau^-^, Aβ^+^/tau^-^, and Aβ^+^/tau^+^ of all iNPH patients (n=68). Points colored in gray represent measurements stemming from iNPH patients with a concomitant neurodegenerative disease. *P*-values were determined using ANOVA.

**Additional file 3: Table S1.** Parameters of the Spearman rank-order correlation of (i) the abundance of the top four biomarker candidates with corresponding improvement on the iNPHGS one-year post-shunting (blue) and (ii) the abundance of the top five biomarkers with change in gait speed one year after surgery (green). iNPH patients with comorbid neurodegenerative condition prior shunting were excluded for the analyses. *P*-values were adjusted using Benjamini-Hochberg correction. **Table S2.** iNPH patients without comorbid neurodegenerative condition prior shunting were grouped into shunt-responsive (n=28) and unresponsive (n=27) according to their clinical improvement on the iNPHGS one year after shunt installation. To determine significant differences in protein abundance between shunt-responsive and shunt-unresponsive patients, an ANCOVA analysis was performed including age and sex as covariates. Further, the log_2_-transformed fold-change in abundance between both groups was calculated. *P*-values were adjusted using Benjamini-Hochberg correction. **Table S3.** Cross-correlation parameters of iNPHGS, MMSE, CERAD, and gait speed calculated with Spearman rank-order correlation across iNPH patients with and without comorbid neurodegenerative disease prior shunting (n=68).

## Data Availability

The datasets used and/or analysed during the current study are available from the corresponding author on reasonable request.

## Abbreviations

ACN: Acetonitrile
AD: Alzheimer’s disease
ANCOVA: Analysis of covariance
ANXA4: Annexin A4
AUC: Area under the curve
Aβ_1-42_: Amyloid-β 1–42
B3GAT2: Glyceraldehyde-3-phosphate dehydrogenase
CERAD: Consortium to establish a registry for Alzheimer’s disease
CI: Confidence interval
CT: Computer tomography
CSF: Cerebrospinal fluid
CV: Coefficient of variation
DOC: Sodium deoxycholate
DSG2: Desmoglein-2
FA: Formic acid
FABP3: Fatty acid-binding protein, heart
FAIMS: Field asymmetric waveform ion mobility spectrometry
FC: Fold change
FDR: False discovery rate
HCl: Hydrochloric acid
iNPH: Idiopathic normal pressure hydrocephalus
iNPHGS: Idiopathic normal pressure hydrocephalus grading scale
ITGB1: Integrin beta-1
LC–MS: Liquid chromatography couped to mass spectrometry
MCID: Minimal clinically important difference
MIF: Macrophage migration inhibitory factor
MMSE: Mini-Mental State Examination
MRI: Magnetic resonance imaging
OLFM2: Noelin-2
p-tau: Phosphorylated-tau
ROC: Receiver operator characteristics
SPE: Solid phase extraction
TCEP: Tris(2)-carboxyethylphosphine
TFA: Trifluoroacetic acid
TGFBI: Transforming growth factor-beta-induced protein ig-h3
TMT: Tandem mass tag
t-tau: Total-tau
YWHAG: 14-3-3 Protein gamma
