# Supplementary figures and images for: Novel cerebrospinal fluid biomarkers correlating with shunt responsiveness in patients with idiopathic normal pressure hydrocephalus
Source: Fluids Barriers CNS. 2023 Jun 5;20:40. doi: 10.1186/s12987-023-00440-5 (PMC10243080; doi:10.1186/s12987-023-00440-5)

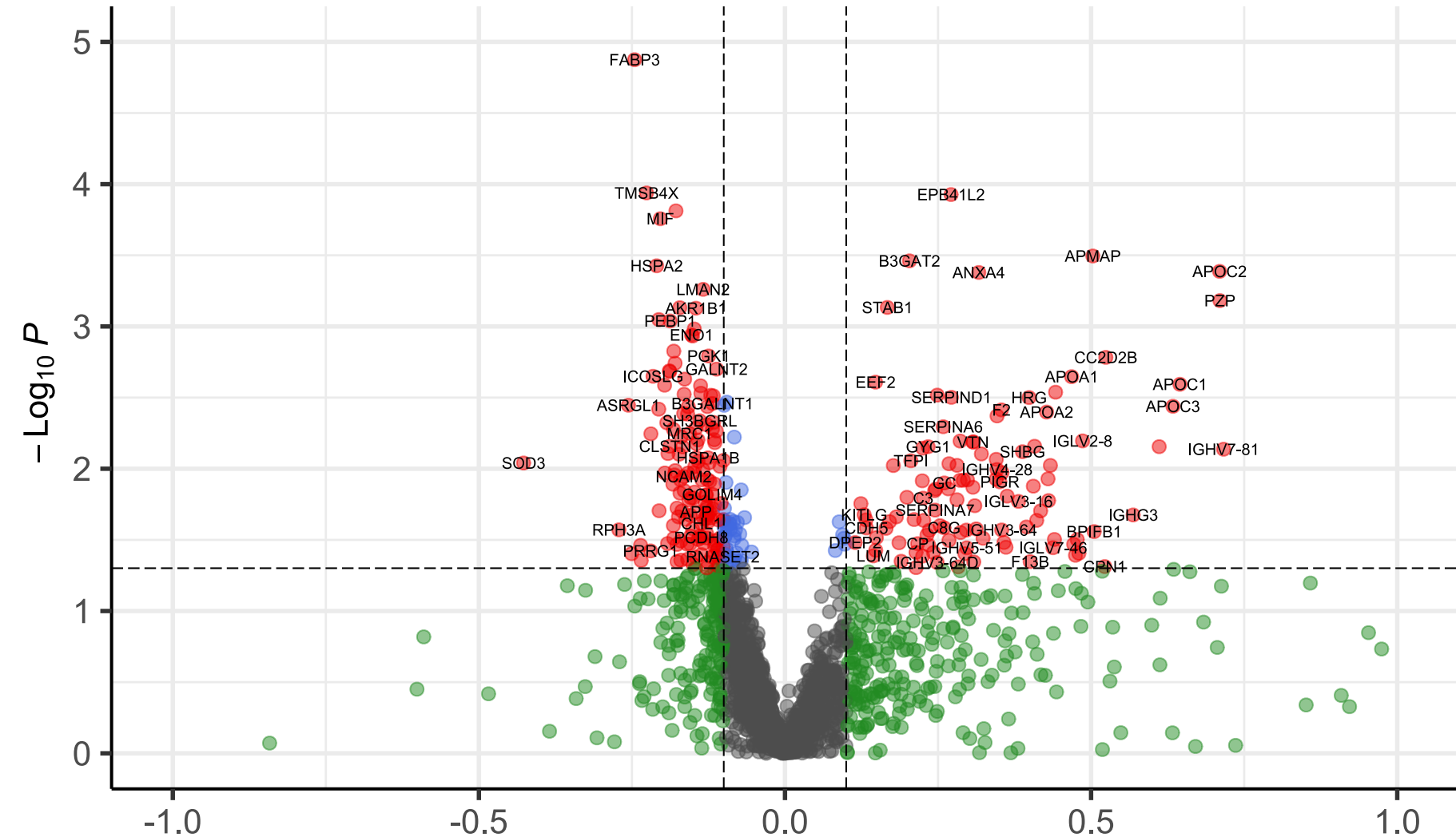

Supplement: Supplementary file 1 — Additional file 1: Figure S1. Volcano plot comparing CSF protein abundances of shunt-responsive (n=35) and shunt-unresponsive iNPH patients (n=33) one-year post-shunting. Log2-fold change (FC) cut-off: 0.1; p-value cut-off: 0.05. The p-values were not FDR-corrected. [file 12987_2023_440_MOESM1_ESM.pdf]

associated with iNPHGS

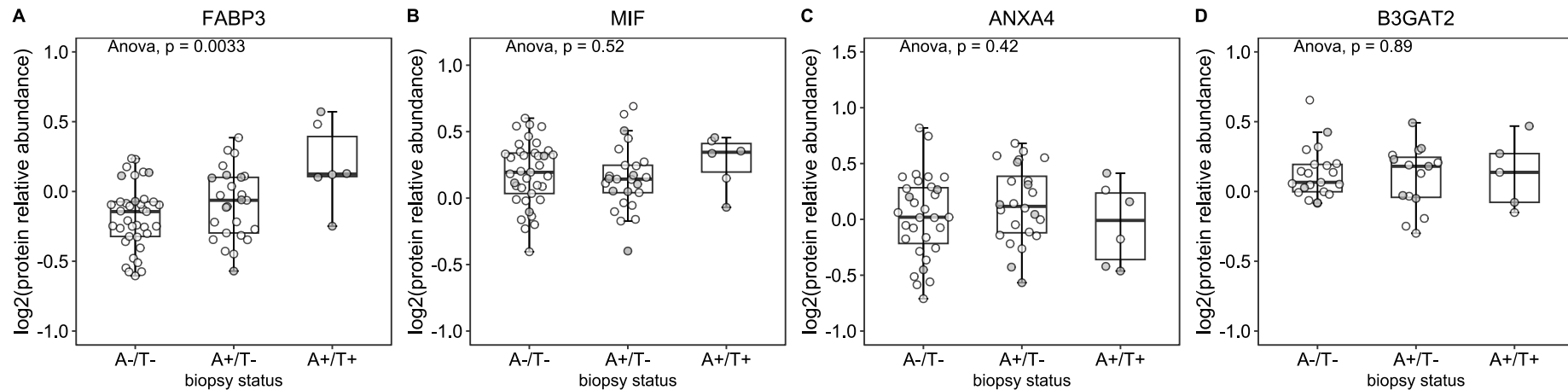

associated with gait velocity

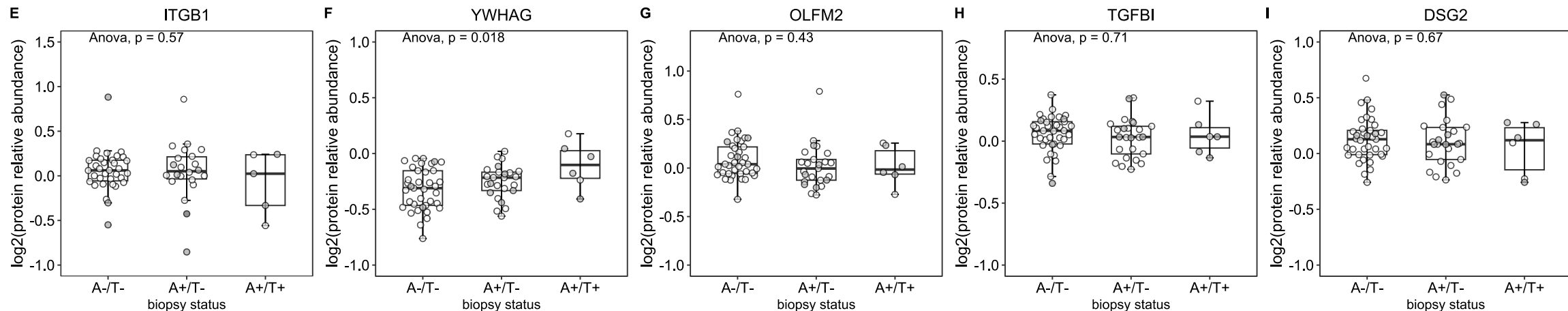

Supplement: Supplementary file 2 — Additional file 2: Figure S2. Log2-transformed protein abundance of the biomarker candidates FABP3 (A), MIF (B), ANXA4 (C) and B3GAT2 (D), ITGB1 (E), YWHAG (F), OLFM2 (G), TGFBI (H), and DSG (I) across the biopsy status groups Aβ-/tau-, Aβ+/tau-, and Aβ+/tau+ of all iNPH patients (n=68). Points colored in gray represent measurements stemming from iNPH patients with a concomitant neurodegenerative disease. P-values were determined using ANOVA. [file 12987_2023_440_MOESM2_ESM.pdf]
