# Supplementary material for: Novel cerebrospinal fluid biomarkers correlating with shunt responsiveness in patients with idiopathic normal pressure hydrocephalus
Source: Fluids Barriers CNS. 2023 Jun 5;20:40. doi: 10.1186/s12987-023-00440-5 (PMC10243080; doi:10.1186/s12987-023-00440-5)
Supplement: Supplementary file 3 — Additional file 3: Table S1. Parameters of the Spearman rank-order correlation of (i) the abundance of the top four biomarker candidates with corresponding improvement on the iNPHGS one-year post-shunting (blue) and (ii) the abundance of the top five biomarkers with change in gait speed one year after surgery (green). iNPH patients with comorbid neurodegenerative condition prior shunting were excluded for the analyses. P-values were adjusted using Benjamini-Hochberg correction. Table S2. iNPH patients without comorbid neurodegenerative condition prior shunting were grouped into shunt-responsive (n=28) and unresponsive (n=27) according to their clinical improvement on the iNPHGS one year after shunt installation. To determine significant differences in protein abundance between shunt-responsive and shunt-unresponsive patients, an ANCOVA analysis was performed including age and sex as covariates. Further, the log2-transformed fold-change in abundance between both groups was calculated. P-values were adjusted using Benjamini-Hochberg correction. Table S3. Cross-correlation parameters of iNPHGS, MMSE, CERAD, and gait speed calculated with Spearman rank-order correlation across iNPH patients with and without comorbid neurodegenerative disease prior shunting (n=68). [file 12987_2023_440_MOESM3_ESM.docx]

**Supplementary table S1**: Parameters of the Spearman rank-order correlation of (i) the abundance of the top four biomarker candidates with corresponding improvement on the iNPHGS one-year post-shunting (blue) and (ii) the abundance of the top five biomarkers with change in gait speed one year after surgery (green). iNPH patients with comorbid neurodegenerative condition prior shunting were excluded for the analyses. *P*-values were adjusted using Benjamini-Hochberg correction.

| Accession | Gene Symbol | Spearman correlation coefficient *R* | *p*-value | FDR-adjusted *p*-value |
| --- | --- | --- | --- | --- |
| P05413 | FABP3 | -0,47 | <0.001 | 0,099 |
| P14174 | MIF | -0,47 | <0.001 | 0,099 |
| P09525 | ANXA4 | 0,47 | <0.001 | 0,142 |
| Q9NPZ5 | B3GAT2 | 0,65 | <0.001 | 0,094 |
| P05556 | ITGB1 | -0,46 | <0.01 | 0,834 |
| P61981 | YWHAG | -0,43 | <0.01 | 0,834 |
| O95897 | OLFM2 | 0,36 | <0.05 | 0,877 |
| Q15582 | TGFBI | -0.40 | <0.01 | 0,877 |
| Q14126 | DSG2 | 0.41 | <0.01 | 0,877 |

**Supplementary table S2**: iNPH patients without comorbid neurodegenerative condition prior shunting were grouped into shunt-responsive (n=28) and unresponsive (n=27) according to their clinical improvement on the iNPHGS one year after shunt installation. To determine significant differences in protein abundance between shunt-responsive and shunt-unresponsive patients, an ANCOVA analysis was performed including age and sex as covariates. Further, the log_2_-transformed fold-change in abundance between both groups was calculated. *P*-values were adjusted using Benjamini-Hochberg correction.

| Accession | Gene Symbol | *p*-value | FDR-adjusted *p*-value | log_2_(fold change) |
| --- | --- | --- | --- | --- |
| P05413 | FABP3 | <0.001 | 0,099 | -0,23 |
| P14174 | MIF | <0.001 | 0,118 | -0,21 |
| P09525 | ANXA4 | <0.01 | 0,170 | 0,29 |
| Q9NPZ5 | B3GAT2 | <0.01 | 0,151 | 0,22 |

**Supplementary table S3**: Cross-correlation parameters of iNPHGS, MMSE, CERAD, and gait speed calculated with Spearman rank-order correlation across iNPH patients with and without comorbid neurodegenerative disease prior shunting (n=68).

| comparison | time point | Spearman correlation coefficient *R* | *p*-value |
| --- | --- | --- | --- |
| iNPHGS-CERAD | baseline | -0.25 | 0.034 |
| iNPHGS-CERAD | 3-months follow-up | -0.58 | <0.001 |
| iNPHGS-CERAD | 1-year follow-up | -0.49 | <0.001 |
| iNPHGS-MMSE | baseline | -0.36 | <0.001 |
| iNPHGS-MMSE | 3-months follow-up | -0.45 | <0.001 |
| iNPHGS-MMSE | 1-year follow-up | -0.59 | <0.001 |
| iNPHGS-gait | baseline | -0.50 | <0.001 |
| iNPHGS-gait | 3-months follow-up | -0.58 | <0.001 |
| iNPHGS-gait | 1-year follow-up | -0.59 | <0.001 |
| CERAD-MMSE | baseline | 0.75 | <0.001 |
| CERAD-MMSE | 3-months follow-up | 0.65 | <0.001 |
| CERAD-MMSE | 1-year follow-up | 0.75 | <0.001 |
